# Associations between Dietary Factors and Self-Reported Physical Health in Chinese Scientific Workers

**DOI:** 10.3390/ijerph121215041

**Published:** 2015-12-18

**Authors:** Qian-fen Gong, Ling Tu, Liang Zhou, Hong Chen

**Affiliations:** 1 Department of Nutrition, Southwest Hospital, Third Military Medical University, Chongqing 400038, China; tuling1982@163.com (L.T.); Hong1983@sohu.com (H.C.); 2 Department of Health Statistics, College of Preventive Medicine, Third Military Medical University, Chongqing 400038, China; zliang1986@sohu.com

**Keywords:** dietary factors, SF-36, health-related quality of life, scientific workers

## Abstract

*Background*: Scientific workers play an important role in the development of science and technology. However, evidence is lacking with regard to the associations between their dietary factors and their health-related quality of life (HRQOL). *Methods*: A cross-sectional survey was conducted among 775 scientific workers from multiple universities and institutes in the Southwest region of China. A self-administered food-frequency questionnaire was used to collect the food consumption information, and the 36-item Short-Form Health Survey was used to assess physical HRQOL. Hierarchical multiple regression analysis was used to identify the factors associated with scientific workers’ HRQOL. *Results*: Physical HRQOL was negatively associated with age and intake of fresh pork (fat) and animal viscera, whereas consumption of vegetables, fruits, refined cereals and dairy products were positively correlated with physical HRQOL. Participants with daily intake of vegetable oils or mixed oils showed higher physical HRQOL scores than those with intake of animal oils. *Conclusions*: Dietary habits are closely associated with the physical HRQOL of scientific workers. The dietary patterns that had more vegetables and fruits, less fresh pork (fat) and animal viscera, and used vegetable oils during cooking corresponded to higher physical HRQOL scores. These findings are important for planning dietary strategies to improve physical health in scientific workers.

## 1. Introduction

Scientific workers, defined as persons whose major professional activity is to conduct scientific research, play a large role in the rapid development of science and technology. Scientific workers experience high occupational stress, irregular lifestyles, increased cognitive demands, and decreased physical activity. These problems threaten their health related quality of life (HRQOL), which is concerning for both the government and the general population. Staying healthy, both mentally and physically, is an important but difficult topic not only in the general public but also in occupational populations worldwide. Numerous efforts have been made to explore the factors related to HRQOL, including social factors [1], physical factors [2], psychological factors [3], and nutritional factors [4].

HRQOL, which was developed to assess a person’s health status, is an individual’s satisfaction or happiness as measured by a multidimensional concept referring to the physical, psychological and social domains of health. Many questionnaires have been used to evaluate HRQOL to date. Among them, the 36-item Short Form Health Survey (SF-36) is widely considered to be the most common [5]. The questionnaire consists of 36 items in eight dimensions of health, each of which has different components that evaluate both subjective (perceptions) and objective (functioning and health status) dimensions of health. HRQOL measurements have been frequently applied to patients, but they are also used among healthy populations. There are several well-known determinants of HRQOL [6], some of which can be modified because they are based on individual behaviors [7]. Among these behavioral determinants, dietary habits have attracted great interest from scientists and the general public. 

The application of dietary patterns has been of particular interest in the field of nutritional epidemiology [8]. In healthy populations, dietary patterns appear to be associated with different health conditions. Results from a cohort study showed that participants with the highest baseline diet scores had higher adjusted mean scores in several SF-36 domains 5 years later, including physical function, general health, vitality, and physical composite score. Therefore, higher diet quality is prospectively associated with better quality of life and functional ability [9]. Another cohort study demonstrated that baseline adherence to a Western dietary pattern was inversely associated with self-perceived quality of life after 4 years of follow-up, whereas baseline adherence to a Mediterranean dietary pattern was positively associated with quality of life scores four years later [10]. Moreover, in a population-based cross-sectional study, increasing daily intake of fruit and vegetables by two portions has been shown to be associated with an 11% higher likelihood of good functional health. Thus, higher fruit and vegetable consumption is associated with better self-reported physical functional health within a general population [11]. Moreover, dietary pattern changes are highly associated with HRQOL in patients with cancer [12,13], inflammatory bowel disease [14], celiac disease [15], meibomian gland dysfunction [16], and others. 

However, to our knowledge, there is a lack of evidence regarding the dietary factors affecting HRQOL among Chinese scientific workers. Accordingly, we conducted the present cross-sectional study to assess the associations between dietary intake and HRQOL in Chinese scientific workers and to explore its predictive factors. This study may provide evidence and a theoretical basis for developing strategies to improve QOL in Chinese scientific workers.

## 2. Materials and Methods

### 2.1. Ethics Statement

The study protocol was approved by the ethical committee on human experimentation of the Third Military Medical University, Chongqing, China (2014(133)). Interviewers read consent forms to the participants and participants signed to give informed consent.

### 2.2. Study Design and Sample

Data used in this cross-sectional study came from a baseline survey of 775 scientific workers from multiple universities or institutes in Southwest China, including the Third Military Medical University, Chongqing Medical University, Chongqing University, and the China Academy of Engineering Physics. The participants included 585 men and 190 women, aged from 30 to 65 years old. Eligibility criteria of this population included the following: (1) at least five years of experience in scientific research; (2) absence of any chronic diseases, such as hypertension, diabetes, *etc.*; and (3) clinically proven absence of cancer. Participants with no documentation, or incomplete documentation, of these criteria were excluded. 

### 2.3. Measurements of General Characteristics

General characteristics included gender, age in years, body mass index (BMI), educational level, monthly income, marital status, smoking, drinking, exercise, work/rest cycle and sleep quality. Age was categorized as ≤45, 46–60 and >60 years old. BMI was calculated from the values of body weight (kg) divided by the square of body length (m^2^) and was categorized into three groups: Normal (18.5–23.9), Overweight (24–28), and Obese (>28). Educational level was divided into Bachelor’s or lower, Master’s, and Doctoral. Monthly income was categorized as <5000, 5000–10,000 and >10,000 Yuan. Marital status was either “Married” or “Unmarried or Divorced”. Responses to the Smoking and Drinking variables were either Yes or No. Participant level of exercise was categorized as Never, Once per week, and Twice per week or more. Work/rest cycle was defined as Regular or Irregular according to the regularity of the participants’ Work/rest cycle. Sleep quality was categorized as Good and Poor. Poor sleep quality was defined as meeting any of the following criteria: <5 h of sleep per night, insomnia, use of sleeping medication, or daytime dysfunction.

### 2.4. Dietary Intake Assessment

Food consumption information was collected by a modified validated self-administered food-frequency questionnaire (FFQ), which assessed self-reported intake of food in the previous 12 months [17]. The validity and reproducibility of this questionnaire have been evaluated in a previous study [18]. The FFQ included eighty-one food items and covered most of the commonly consumed foods in the southwest of China. The survey included nine validated short questions on food habits concerning: (1) refined cereals, including rice, pasta, white bread, cold breakfast cereals, 1 unit = 250 g; (2) legumes and tubers, including lentils, chickpeas, beans, peas, potatoes, sweet potatoes and yam, 1 unit = 50 g; (3) dairy products, including whole milk, condensed milk, yogurt, custard, cream, milk shake, cheese, or other dairy products, 1 unit = 250 mL; (4) fresh pork (lean), 1 unit = 100 g; (5) fresh pork (fat), 1 unit = 100 g; (6) animal viscera, including the brain, liver, kidney, heart, stomach, or intestines of cattle, pig, sheep or other animals, 1 unit = 100 g; (7) vegetables, including tomatoes, carrots, cauliflower, lettuce, green beans, eggplant, swiss chard, peppers, asparagus, spinach and other fresh vegetables, 1 unit = 500 g; (8) fruits, including banana, pear, melon, watermelon, citrus, strawberry, peach, cherry, fig, grapes, kiwi, mango, 1 unit = 300 g, and (9) cooking oils, including three dimensions: animal oils, such as butter, lard, vegetable oils, such as salad oil, sunflower oil, corn oil, nut oils, spray oils, margarines, or olive oil, and mixed oils, both animal and vegetable oils that were used in daily cooking. For each food item or food group, participants were asked how frequently (daily, weekly, monthly) they consumed the food or food group, followed by a question on the amount of consumption in units over the past 12 months.

### 2.5. Measurements of Physical HRQOL

The 36-item Short-Form Health Survey (SF-36) was used to measure physical HRQOL. The Mandarin version of the SF-36 has been shown to be a valid and reliable assessment of quality of life with a Cronbach’s alpha ranging from 0.75 to 0.90 for the eight dimensions [5]. Therefore, the SF-36 was used to assess quality of life in this study. This instrument contained 36 items and measured eight dimensions of HRQOL: physical functioning (PF), role-physical (RP), bodily pain (BP), general health (GH), vitality (VT), social functioning (SF), role limitation due to emotional problems (RE), and mental health (MH). PF, RP, BP and GH scores were used to create the physical component summary (PCS); a score was calculated for each dimension and was transformed to obtain a value ranging from 0 to 100, with higher scores indicating better health [19]. These five parameters (PF, RP, BP, GH and PCS) were described in detail and further analyzed to explore the dietary factors associated with physical HRQOL in our study.

### 2.6. Statistical Analysis

SPSS 18.0 statistical program (SPSS Inc., Chicago, IL, USA) was used to perform two sample t-test, one-way ANOVA or Kruskal-Wallis H test, and multivariate stepwise regression to evaluate the influencing factors on quality of life. The distributions of HRQOL in categorical variables were evaluated using t-test and ANOVA. If equal variances were not assumed when compared the difference between two samples, t- test was used to weight data to reduce variances. Whereas one-way ANOVA was used in the comparison among multiple variables, if equal variances were not assumed, Kruskal-Wallis H test was used to conduct this comparison. The correlation between HRQOL scores and continuous variables was assessed by Pearson’s correlation. If the correlation between two variables was more than 0.5, these variables were regarded as co-line variables. In this study, no co-line variables were found, multivariate stepwise regression were then performed using each cluster of HRQOL as dependent variables, and general characteristics as well as dietary factors as independent variables. A *p* < 0.05 was considered statistically significant, results were presented as means ± standard deviations (SD).

## 3. Results

### 3.1. Basic Characteristics of Participants

Data were obtained from 775 scientific workers. The basic characteristics of the participants are provided in Table 1. The age of participants ranged from 30 to 65 years. Most participants were men (*N* = 585, 75.5%). The majority of participants had a normal BMI (*N* = 564, 72.8%), 155 participants were overweight, and 56 participants were obese. Of the 775 participants, 35.6% (*N* = 276) had a Doctoral degree, 27.0% (N = 209) had a Master’s degree, and the rest of the participants (*N* = 290) had a bachelor’s degree or did not have any degree. The monthly income of 49.0% of the participants (*N* = 380) was lower than 5000 Yuan; just 9.3% of participants (*N* = 72) earned more than 10,000 Yuan every month. Regarding participant marital status, the majority (79.7%) of participants were married and 20.3% were single or divorced. A number of participants were smokers (*N* = 452, 58.3%) or drinkers (*N* = 164, 21.2%). With respect to their exercising habits, 30.7% of participants (*N* = 238) never exercised, 43.1% of participants (*N* = 334) exercised once per week, and 26.2% participants (*N* = 203) exercised twice or more per week. Four hundred fifty-three participants (58.5%) had a regular work/rest cycle, whereas the rest had an irregular cycle, and 314 participants (40.5%) had good sleep quality, while the rest did not. More baseline characteristics of participants are presented in Table 1.

**Table 1:** PF, RP, BP, GH and PCS scores based on the characteristics of scientific workers (Mean ± SD).

Variable	N	PF	RP	BP	GH	PCS
Gender						
Male	585	82.63 ± 18.22 *	83.88 ± 19.95	76.84 ± 19.38 *	60.26 ± 19.08	75.90 ± 14.17 *
Female	190	77.25 ± 16.68 *	83.80 ± 17.87	72.43 ± 20.48 *	59.69 ± 19.08	73.29 ± 13.97 *
Age						
≤45	226	89.27 ± 15.49 *	86.12 ± 17.85 *	80.53 ± 17.05 *	68.19 ± 18.95 *	81.03 ± 12.85 *
46–60	385	80.64 ± 16.16 *	83.83 ± 19.45 *	75.20 ± 19.87 *	58.15 ± 18.21 *	74.45 ± 13.39 *
>60	164	72.04 ± 20.37 *	80.90 ± 21.22 *	70.49 ± 21.33 *	53.68 ± 17.54 *	69.28 ± 14.73 *
BMI (kg/m^2^)						
Normal (18.5–23.9)	564	82.61 ± 17.53 *	84.08 ± 19.23	75.73 ± 19.69	61.41 ± 19.08 *	75.95 ± 14.13 *
Overweight (24–28)	155	79.45 ± 17.65 *	84.92 ± 19.41	76.21 ± 19.70	57.75 ± 18.23 *	74.58 ± 13.61 *
Obese (>28)	56	73.64 ± 21.33 *	79.02 ± 21.33	74.84 ± 20.42	53.84 ± 19.56 *	70.36 ± 15.07 *
Educational level						
Bachelor or lower	290	83.57 ± 16.48 *	85.53 ± 20.01	75.80 ± 19.33	64.75 ± 18.76 *	77.41 ± 13.32 *
Master’s	209	83.16 ± 17.75 *	84.75 ± 18.28	77.37 ± 20.24	60.00 ± 18.68 *	76.32 ± 14.32 *
Doctoral	276	80.02 ± 17.46 *	82.84 ± 20.49	75.36 ± 19.38	58.47 ± 18.61 *	74.17 ± 13.58 *
Monthly income						
<5000 Yuan	380	81.88 ± 18.65	84.26 ± 19.75	76.24 ± 19.94	61.48 ± 18.78	75.97 ± 14.43
5000–10,000 Yuan	323	80.53 ± 16.94	83.50 ± 19.39	74.63 ± 19.35	58.83 ± 18.73	74.39 ± 13.72
>10,000 Yuan	72	82.08 ± 19.15	83.59 ± 18.36	78.29 ± 20.12	58.39 ± 21.61	75.59 ± 14.54
Marital status						
Married	618	81.26 ± 17.68	83.82 ± 19.57	75.76 ± 19.62	59.92 ± 19.15	75.19 ± 14.11
Unmarried/Divorced	157	81.62 ± 19.23	84.12 ± 19.02	75.77 ± 20.19	60.97 ± 19.76	75.62 ± 14.35
Smoking						
No	323	80.84 ± 19.06	83.48 ± 19.49	73.76 ± 21.01	59.01 ± 18.83	74.27 ± 15.07
Yes	452	80.12 ± 18.04	85.52 ± 18.93	74.88 ± 14.13	59.24 ± 17.66	74.94 ± 11.70
Drinking						
No	611	81.24 ± 18.36	83.42 ± 19.51	75.54 ± 19.97	59.38 ± 19.34	74.89 ± 14.35
Yes	164	82.20 ± 15.58	84.55 ± 19.72	76.85 ± 18.01	62.70 ± 17.47	76.57 ± 12.66
Exercise						
Never	238	80.59 ± 18.20	82.46 ± 20.54	72.23 ± 20.20 *	58.81 ± 18.96	73.52 ± 14.19 *
Once per week	334	82.44 ± 16.71	84.69 ± 18.72	78.24 ± 19.07 *	61.01 ± 18.69	76.60 ± 13.58 *
Twice per week or more	203	83.39 ± 19.70	84.21 ± 19.32	75.82 ± 19.66 *	60.23 ± 19.77	75.16 ± 14.86 *
Work/rest cycle						
Regular	453	81.28 ± 17.87	84.26 ± 18.78	75.83 ± 19.43	59.96 ± 19.48	75.33 ± 13.70
Irregular	321	80.84 ± 18.54	83.18 ± 20.35	75.30 ± 20.25	59.71 ± 18.74	74.76 ± 14.77
Sleep quality						
Good	314	81.40 ± 17.34	84.28 ± 18.39	74.89 ± 19.09	59.39 ± 19.34	74.99 ± 13.83
Poor	412	80.73 ± 19.40	83.42 ± 20.51	76.25 ± 20.41	59.74 ± 18.89	75.04 ± 14.86

Notes: * *p* < 0.05, BMI: body mass index, PF: physical functioning, RP: role-physical, BP: bodily pain, GH: general health, PCS: physical component summary, SD: standard deviation.

### 3.2. Description of Physical HRQOL

In this study, mean ± SD scores of PF, RP, BP, GH and PCS based on the characteristics of participants are shown in Table 1. There were gender differences in RF, BP and PCS scores; male scientific workers had higher scores than their female counterparts. Scores of PF, RP, BP, GH and PCS significantly decreased with age, especially in the population above 60 years old. Scientific workers with a higher BMI had lower scores in PF, GH and PCS. As for educational level, although there were no differences between Bachelor’s or lower and Master’s levels, scientific workers with Doctoral degrees had lower PF, GH and PCS scores. Furthermore, participants who regularly exercised scored higher in PF and PCS than those who never exercised. However, significant differences in monthly income, marital status, smoking, drinking, work/rest cycle and sleep quality were not found. Because there was no difference of PF, RP, BP, GH and PCS scores in work/rest cycle and sleep quality, samples who just only omit the information of work/rest cycle and sleep quality have not been excluded, so that there were missing sample sizes for work/rest cycle and sleep quality.

Table 2 shows the physical HRQOL outcomes in scientific workers with different dietary patterns. Scientific workers who consumed more than 1 unit of refined cereals or fresh pork (lean) per day had significantly higher scores in PF and RP, but not BP, GH, or PCS, compared with workers consuming 1–2 units of refined cereal or lean pork per month. Moreover, participants with higher rates of legumes and tubers consumption had higher RP, BP, and PCS scores. A higher frequency of dairy product, vegetable, and fruit intake also resulted in higher PF, BP, GH and PCS scores. As for the cooking oils, participants with intake of vegetable oils or mixed oils scored higher in RP, BP and PCS than those who used animal oils. However, participants with a higher frequent consumption of fresh pork (fat) and animal viscera had lower scores in PF, RP, PCS, and/or BP, and GH.

**Table 2:** Scores of PF, RP, BP, GH and PCS based on dietary factors of scientific workers (Mean ± SD).

Variable	N	PF	RP	BP	GH	PCS
Refined cereals						
1–2 units/month	56	78.84 ± 22.64 *	80.58 ± 23.77 *	73.86 ± 16.84	61.34 ± 17.78	73.65 ± 14.56
1–2 units/week	267	80.26 ± 17.96 *	82.26 ± 19.67 *	74.64 ± 19.20	60.85 ± 19.42	74.50 ± 14.47
>1 unit/day	396	82.91 ± 16.72 *	86.03 ± 17.80 *	77.00 ± 20.01	60.26 ± 18.82	76.55 ± 13.81
Legumes and tubers						
1–2 units/month	387	82.50 ± 16.89	83.37 ± 19.18 *	75.01 ± 18.83 *	60.43 ± 18.77	75.32 ± 13.50 *
1–2 units/week	182	82.64 ± 15.99	88.53 ± 17.36 *	78.30 ± 19.90 *	61.14 ± 18.79	77.65 ± 13.68 *
>1 unit/day	111	79.32 ± 20.94	81.36 ± 19.92 *	73.79 ± 20.93 *	60.81 ± 20.07	73.82 ± 16.37 *
Dairy products						
1–2 units/month	191	75.26 ± 22.52 *	83.18 ± 19.81	73.10 ± 19.53 *	59.43 ± 19.11 *	72.75 ± 15.23 *
1–2 units/week	226	79.45 ± 16.43 *	84.57 ± 18.31	75.18 ± 19.58 *	56.50 ± 16.65 *	73.92 ± 13.31 *
>1 unit/day	288	87.65 ± 17.80 *	85.13 ± 18.46	77.84 ± 19.29 *	64.46 ± 19.10 *	78.70 ± 13.46 *
Fresh pork (lean)						
1–2 units/month	66	77.35 ± 20.63 *	79.83 ± 22.11 *	75.12 ± 19.02	59.55 ± 17.84	72.96 ± 14.64
1–2 units/week	227	82.69 ± 16.70 *	84.22 ± 19.31 *	75.45 ± 18.60	60.35 ± 17.09	75.68 ± 13.56
>1 unit/day	404	82.26 ± 17.28 *	85.83 ± 17.83 *	76.44 ± 20.03	61.01 ± 20.16	76.38 ± 14.27
Fresh pork (fat)						
1–2 units/month	347	87.62 ± 14.69 *	85.84 ± 17.86 *	78.28 ± 18.32 *	65.11 ± 18.14 *	79.21 ± 12.61 *
1–2 units/week	142	79.82±15.53 *	79.95 ± 20.47 *	75.17 ± 19.15 *	58.10 ± 17.20 *	74.91 ± 12.58 *
>1 unit/day	167	72.63 ± 20.33 *	79.95 ± 20.47 *	71.22 ± 20.82 *	53.21 ± 19.63 *	69.26 ± 15.73 *
Animal viscera						
1–2 units/month	582	82.49 ± 16.44 *	85.02 ± 18.42 *	76.28 ± 18.95	60.88 ± 18.94	76.17 ± 13.67 *
1–2 units/week	51	82.55 ± 19.58 *	83.58 ± 21.79 *	76.55 ± 21.00	60.51 ± 19.82	75.80 ± 15.32 *
>1 unit/day	17	71.76 ± 24.23 *	71.32 ± 20.62 *	67.82 ± 18.87	51.82 ± 20.69	65.70 ± 18.03 *
Vegetables						
1–2 units/month	46	74.89 ± 24.21 *	69.70 ± 23.50 *	74.28 ± 21.85	58.46 ± 19.99 *	69.33 ± 17.87 *
1–2 units/week	58	75.09 ± 21.16 *	82.87 ± 18.72 *	73.84 ± 19.30	52.47 ± 19.81 *	71.07 ± 16.23 *
>1 unit/day	596	82.96 ± 16.21 *	85.61 ± 18.21 *	76.22 ± 19.23	61.52 ± 18.79 *	76.58 ± 13.42 *
Fruits						
1–2 units/month	123	79.51 ± 20.45	77.79 ± 22.49 *	74.67 ± 19.07	56.98 ± 18.16 *	72.24 ± 15.26 *
1–2 units/week	247	82.89 ± 15.59	84.51 ± 18.55 *	77.21 ± 18.34	59.74 ± 19.20 *	76.10 ± 13.39 *
>1 unit/day	345	81.52 ± 18.34	86.47 ± 17.55 *	75.51 ± 20.30	62.40 ± 19.00 *	76.48 ± 14.16 *
Cooking Oils						
Animal oils	11	75.91 ± 21.66	72.16 ± 32.04 *	72.36 ± 17.39 *	57.45 ± 9.40	69.47 ± 16.95 *
Vegetable oils	589	81.14 ± 18.03	83.72 ± 19.39 *	74.64 ± 19.70 *	59.85 ± 18.86	74.84 ± 13.98 *
Mixed oils	146	82.43 ± 18.03	85.10 ± 18.09 *	80.21 ± 19.34 *	62.08 ± 19.87	77.46 ± 14.21 *

Notes: * *p* < 0.05, PF: physical functioning, RP: role-physical, BP: bodily pain, GH: general health, PCS: physical component summary, SD: standard deviation.

### 3.3. Correlations between Variables and Predictors of Physical HRQOL

The results of the hierarchical multiple regression analysis of PF, RP, BP, GH and PCS are presented in Table 3, Table 4, Table 5, Table 6 and Table 7. In the regression model, age was the only demographic characteristic of scientific workers that significantly correlated with physical HRQOL, whereas the dietary factors that affected the physical HRQOL scores included refined cereals, fresh pork (fat), dairy products, vegetables, fruits, animal viscera, and cooking oils. Dietary intake of fresh pork (fat) and animal viscera as well as participants’ age were negatively associated with PF, whereas intake of dairy products and fruits were positively associated with PF (Table 3). Consumption of vegetables and refined cereals was positively correlated with RP, whereas animal viscera consumption and age were negatively associated with RP (Table 4). BP scores were negatively associated with age, but positively associated with the types of cooking oils used (Table 5). Age and fresh pork (fat) intake showed negative associations with GH, whereas fruit consumption was positively correlated with GH (Table 6). PCS, which was the sum of PF, RP, BP and GH, was significantly negatively associated with age and intake of fresh pork (fat) and animal viscera, but positively correlated with vegetable and fruit consumption (Table 7).

**Table 3:** Stepwise regression predicting the PF scores.

Variable	Physical Functioning (PF)					
	Pearson’s r	Step 1 (β)	Step 2 (β)	Step 3 (β)	Step 4 (β)	Step 5 (β)
Fresh pork (fat)	0.358	−7.056 ***	−6.323 ***	−4.772 ***	−5.313 ***	−4.950 ***
Dairy products	0.418		4.451 ***	4.153 ***	3.631 ***	3.742 ***
Age	0.451			−4.470 ***	−4.070 ***	−4.304 ***
Fruits	0.471				3.466 ***	3.584 ***
Animal viscera	0.478					−3.292 *

Notes: * *p* < 0.05, *** *p* < 0.001.

**Table 4:** Stepwise regression predicting the PF scores.

Variable	Role-Physical (RP)				
	Pearson’s r	Step 1 (β)	Step 2 (β)	Step 3 (β)	Step 4 (β)
Vegetables	0.210	7.134 ***	7.274 ***	7.275 ***	6.813 ***
Animal viscera	0.242		−5.443 **	−5.603 **	−5.696 **
Age	0.268			−3.100 **	−3.117 **
Refined cereals	0.283				2.697 *

Notes: * *p* < 0.05, ** *p* < 0.01, *** *p* < 0.001.

**Table 5:** Stepwise regression predicting the BP scores.

Variable	Body Pain (BP)		
	Pearson’s r	Step 1 (β)	Step 2 (β)
Age	0.204	−5.718 ***	−5.603 ***
Cooking oils	0.219		3.445 *

Notes: * *p* < 0.05, *** *p* < 0.001.

**Table 6:** Stepwise regression predicting the GH scores.

Variable	Physical Component Summary (PCS)					
	Pearson’s r	Step 1 (β)	Step 2 (β)	Step 3 (β)	Step 4 (β)	Step 5 (β)
Age	0.311	−6.199 ***	−6.280 ***	−4.718 ***	−4.998 ***	−5.026 ***
Fruits	0.356		3.125 ***	3.159 ***	3.337 ***	2.743 ***
Fresh pork (fat)	0.392			−2.614 ***	−3.582 ***	−3.565 ***
Animal viscera	0.405				−3.582 ***	−3.565 **
Vegetables	0.415					2.383 *

Note: * *p* < 0.05, ** *p* < 0.01, *** *p* < 0.001.

**Table 7:** Stepwise regression predicting the PCS scores.

Variable	General Health (GH)			
	Pearson’s r	Step 1 (β)	Step 2 (β)	Step 3 (β)
Age	0.316	−8.586 ***	−8.685 ***	−6.788 ***
Fruits	0.367		4.651 ***	4.645 ***
Fresh pork (fat)	0.393			−3.556 ***

Note: *** *p* < 0.001.

## 4. Discussion

In this population-based cross-sectional study, we demonstrated that physical HRQOL was negatively associated with age and consumption of fresh pork (fat) and animal viscera but positively associated with consumption of vegetables, fruits, refined cereals and dairy products. In addition, the types of cooking oils used were found to be associated with BP; participants with daily intake of vegetable oils or mixed oils were more physically healthy than those with intake of animal oils. 

HRQOL may be affected by many factors. To explore the associations between these factors, hierarchical multiple regression analysis was used to identify factors significantly related to physical HRQOL. Gender, age, BMI, educational level with a Doctoral degree, and the frequency of exercise have significant associations with physical HRQOL scores. Although other studies have suggested that these factors were associated with HRQOL [20,21,22], following regression analysis, only age was significantly related to physical HRQOL in our study. This is consistent with other findings on age patterns, with younger people reporting greater HRQOL than older people [23].

In the present study, the dietary factors that were positively correlated to physical HRQOL included vegetables, fruits, refined cereals and dairy products. This dietary pattern is similar to the Mediterranean diet, which is characterized by plant foods, cereals, legumes, fish and olive oil as the main source of nutrition and is widely considered to be a healthy eating pattern related to reduced risk of cardiovascular and neurodegenerative diseases and some cancers [24]. A previous study found that adherence to a Mediterranean diet was associated with a better HRQOL [25]. The results from a cohort study also demonstrated that baseline adherence to a Mediterranean dietary pattern was directly associated with better scores in quality of life four years later in comparison to the Western dietary pattern, which was rich in red meats, processed pastries and fast-food [10]. Our results were consistent with a previous study that found higher fruit and vegetable consumption to be associated with better self-reported physical functional health within a general population [11]. Antioxidants and polyphenols, which are largely present in vegetables and fruits, have been reported to have anti-inflammatory properties and have also been found to play a protective role against cardiovascular diseases and cancer [26]. Moreover, intake of monounsaturated fatty acids, which is the major component in vegetable oil, has been found to be associated with a reduced prevalence of risk factors for major chronic disease [27,28]. Therefore, the higher physical HRQOL scores found in scientific workers who frequently consumed vegetables, fruits and vegetable oil in this study may be attributed to the role of antioxidants and monounsaturated fatty acids in their dietary intake.

Our data also suggested that frequent consumption of fresh pork (fat) and animal viscera, which are rich in lipid, cholestenone and polyunsaturated fatty acids, seemed to be deleterious to quality of life. Both epidemiological and experimental studies have reported a detrimental effect of this dietary habit on weight gain, obesity and diabetes [29,30]. Furthermore, this pattern has also been associated with an increasing risk of cardiovascular diseases, endothelial dysfunction and a higher level of pro-inflammatory cytokines [31,32,33]. The high content of saturated, trans-unsaturated fatty acids usually present in these foods is thought to be responsible for the reported correlations [34]. The adverse effects of saturated, trans-unsaturated fats on cardiovascular diseases are considered to be associated with reductions in plasma levels of high density lipoprotein (HDL)-cholesterol, increases in low density lipoprotein (LDL)-cholesterol, endothelial dysfunction, pro-inflammatory changes, and displacement of essential fatty acids from membranes [35]. These changes may account for the negative associations between frequent consumption of fresh pork (fat) and animal viscera and physical HRQOL in the present study.

Although HRQOL is a subjective health measurement rather than a biological measure, self-reported physical health status has been reported to be a powerful predictor of long-term mortality [36,37]. Although age has been shown to be a significant predictor of physical HRQOL, it may be impossible to reverse. Dietary habits, on the other hand, can be changed to improve physical HRQOL. Therefore, we recommend that it is important for scientific workers to consume more vegetables and fruits, less fresh pork (fat) and animal viscera, and to use vegetable oils during cooking as much as possible to stay healthy. There were some limitations to the present study, which has all the limitations of a cross-sectional study. Firstly, the cross-sectional design did not allow us to make any causal statements about the relationships between the variables investigated. Secondly, although the reliability and validity of the FFQ and SF-36 questionnaire have been widely evaluated, misclassifications may have existed in the dietary and outcomes assessments, meaning over- or underestimation of true intake and health status may have introduced bias during the data analysis. Thirdly, caution is needed in generalizing the conclusions to larger contexts because participants were from just one province and represented only one career. 

## 5. Conclusions

Our data found an association between dietary habits and self-reported physical health status in scientific workers. The dietary patterns that had more vegetables and fruits, less fresh pork (fat) and animal viscera, and used vegetable oils during cooking were linked with higher scores of physical HRQOL. These findings are important for planning dietary strategies to improve physical health in scientific workers. Further investigations should be conducted with a large survey including random samples of scientific workers around the world.
